# Translational Regulators in Pulmonary Fibrosis: MicroRNAs, Long Non-Coding RNAs, and Transcript Modifications

**DOI:** 10.3390/cells14070536

**Published:** 2025-04-03

**Authors:** Sumeen Kaur Gill, Richard H. Gomer

**Affiliations:** Department of Biology, Texas A&M University, College Station, TX 77843, USA; sumeen.gill@tamu.edu

**Keywords:** idiopathic pulmonary fibrosis, microRNA, long non-coding RNA, alternative polyadenylation, epigenetic modifications, translation

## Abstract

Fibrosing disorders including idiopathic pulmonary fibrosis (IPF) are progressive irreversible diseases, often with poor prognoses, characterized by the accumulation of excessive scar tissue and extracellular matrix. Translational regulation has emerged as a critical aspect of gene expression control, and the dysregulation of key effectors is associated with disease pathogenesis. This review examines the current literature on translational regulators in IPF, focusing on microRNAs (miRNAs), long non-coding RNAs (lncRNAs), and RNA transcript modifications including alternative polyadenylation and chemical modification. Some of these translational regulators potentiate fibrosis, and some of the regulators inhibit fibrosis. In IPF, some of the profibrotic regulators are upregulated, and some of the antifibrotic regulators are downregulated. Correcting these defects in IPF-associated translational regulators could be an intriguing avenue for therapeutics.

## 1. Introduction

The regulatory elements that mediate transcription, such as transcription factors, DNA methylation, and histone modifications, have been relatively well characterized for over 50 years [1]. However, recent studies have highlighted the emerging role of translational regulation and RNA modification in gene expression control. Eukaryotic translation is a highly complex and tightly controlled process that involves several key steps after mRNA is exported from the nucleus, including scanning, initiation, elongation, and termination [2]. Each step can be regulated by various proteins and signaling molecules, making them potential sites for dysregulation that contributes to disease pathogenesis.

The dysregulation of gene expression is implicated in many pathologies, including fibrosis, a pathological process characterized by excessive extracellular matrix (ECM) deposition and tissue remodeling, which impairs organ function and may progress to organ failure. Fibrotic disorders can be systemic, with fibrosis impacting multiple organs at once, or can be organ-specific, commonly affecting the heart, lungs, liver, or kidneys [3]. Despite its significant impact and poor prognosis, the underlying mechanisms that drive fibrosis are not yet well understood.

This review discusses the current literature on translational regulation in the context of fibrosis, with an emphasis on the lung-specific variant, idiopathic pulmonary fibrosis (IPF). Our previous review discussed the current state of research on RNA-binding proteins (RBPs) and their role in the translational regulation of pulmonary fibrosis [4], and here we review the role of non-coding RNAs and briefly discuss what is currently known about RNA modifications in the translational regulation of fibrosis. Additionally, we propose a model to explain how dynamic RNA effectors and modifications can fine-tune the fibrosis response, as well as the therapeutic potential of RNA-based therapies for fibrosis. This review aims to highlight new avenues for targeted therapeutic strategies and biomarkers for early intervention to help address the unresolved complexities of fibrotic disorders.

## 2. Mechanisms and Insights into Idiopathic Pulmonary Fibrosis

### 2.1. Profibrotic Signaling Cascades

**Figure 1:**
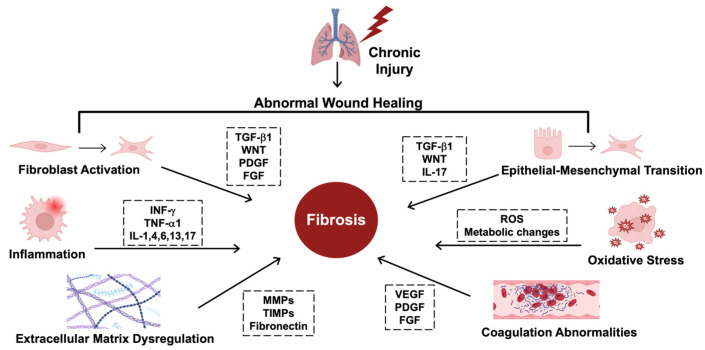
The hallmarks of fibrosing disorders. The mechanisms driving fibrosis are complex and include several key regulators. Starting at the upper left, fibroblast activation and transformation into myofibroblasts is regulated by transforming growth factor beta (TGF-β1), wingless-related integration site (WNT), platelet-derived growth factor (PDGF), and fibroblast growth factor (FGF). Epithelial–mesenchymal transition (EMT), where epithelial cells transform into myofibroblast-like cells, is regulated by TGF-β1, WNT, and interleukin-17 (IL-17). Several key cytokines drive inflammation, including interferon gamma (INF-γ) and tumor necrosis factor (TNF-α1), along with various interleukins, including IL-1, 4, 6, 13, and 17. Due to these transformations, there are also metabolic changes that form reactive oxygen species (ROS) that induce oxidative stress, contributing to fibrosis. Extracellular matrix (ECM) dysregulation includes the overproduction of collagen and disorganized extracellular structures due to abnormalities in the key enzymes that shape the ECM, including matrix metalloproteinases (MMPs), tissue inhibitors of matrix metalloproteinases (TIMPs), and increased fibronectin. Coagulation abnormalities also contribute to fibrosis and are mediated by vascular endothelial growth factor (VEGF), PDGF, and FGF. This figure was created using BioRender elements https://biorender.com/ (accessed on 6 August 2024).

The mechanisms driving fibrosis are complex, encompassing several homeostatic alterations [5]. These processes are coordinated by signaling cascades induced by cytokines, including transforming growth factor beta (TGF-β1), platelet-derived growth factor (PDGF), connective tissue growth factor (CTGF), tumor necrosis factor (TNF-α1), and several interleukins, including IL-1, IL-4, IL-6, and IL-13 [5]. Furthermore, many of these signaling cascades are interconnected. For example, PDGF signaling regulates fibroblast proliferation and differentiation, increasing the production and deposition of collagen while also enhancing the release of TGF-β1 from activated cells [6]. Additionally, downstream regulators of the TGF-β1 signaling cascade can induce CTGF [7]. Similarly, many of the interleukins are interdependent and trigger an inflammatory cascade that leads to processes like EMT and collagen deposition, contributing to fibrosis (Figure 1) [8].

One of the better-studied cytokines in the wound healing and fibrosis pathways is TGF-β1, which is synthesized as a pre-pro-protein monomer with a large N-terminal prodomain called latency-associated peptide (LAP). LAP is required for the proper folding and dimerization of TGF-β1 [9]. After cleavage of the prodomain in the Golgi, LAP and TGF-β1 are secreted and bind to the extracellular matrix. LAP sequesters TGF-β1 and prevents its binding to the extracellular TGF-β1 receptor [9]. Changes in the cellular microenvironment following injury and related events can release active TGF-β1 from LAP [10]. For instance, LAP has an arginine-glycine-aspartic acid (RGD) integrin-binding site, which allows various RGD-binding integrins to release TGF-β1 from LAP [11]. Additionally, the cleavage of the sialic acid groups on LAP by sialidases like neuraminidase 3 (NEU3) can also induce TGF-β1 release [12]. Once released, TGF-β1 binds to a dimer of type I and type II serine/threonine kinase receptors (TβRI and TβRII) on the cells to induce profibrotic signaling cascades through both canonical and noncanonical transcriptionally regulated pathways [13,14,15].

The canonical TGF-β1 pathways are primarily responsible for regulating transcription, mediated by small mothers against decapentaplegic (SMAD) transcription factors [13]. The noncanonical pathways are more diverse, and can be divided into two branches: one that also regulates transcription, including p38 mitogen-activated protein kinase (MAPK) [14], phosphatidylinositol 3-kinase (PI3K), c-Jun N-terminal kinase (JNK), extracellular signal-regulated kinase (ERK), and rat sarcoma virus/rapidly accelerating fibrosarcoma (RAS/RAF) [15]; and another that only regulates translation [16]. In human cardiac fibroblasts, TGF-β1 has an effect on the levels of at least 4216 proteins, and for 33% of these, the change is not due to TGF-β1 affecting the level of the mRNA, but rather TGF-β1 affecting the translation of the mRNA [17]. The mechanisms of this regulation remain largely unexplored. Similarly, the potential roles of other profibrotic cytokines in modulating translation are also poorly understood and require further investigation [17,18].

### 2.2. Hallmarks of IPF

One of the prominent fibrotic disorders is the lung-specific variant, idiopathic pulmonary fibrosis (IPF), which has a poor prognosis, typically resulting in an average survival of 3–5 years following diagnosis [19]. While two drugs, Nintedanib and Pirfenidone, can slow the progression of IPF, the survival of patients remains poor [20]. Understanding the mechanisms of IPF is crucial to develop novel, high-efficacy therapeutics.

While the etiology for IPF remains unclear, several risk factors have been identified, including a history of smoking, exposure to pollutants, occupational hazards, and other exposures that may cause a disruption of various cellular and molecular signals [21]. Notably, chronic lung injury triggers a faulty wound healing response, leading to aberrant gene expression and fibrosis. The classic imaging hallmarks of IPF include the following: (1) honeycomb cysts, which appear as clusters of airspaces with thick walls on chest scans, (2) fibroblastic foci, where a large mass of fibroblasts replaces the normal delicate and lacy tissues in some parts of the lung, and (3) significant thickening of the airway walls and alveolar walls [21]. On a molecular level, the hallmarks of fibrosis include the activation of quiescent fibroblasts into myofibroblasts, which then produce and deposit large amounts of extracellular matrix (ECM) components like collagens and proteoglycans [22]. Epithelial–mesenchymal transition (EMT) also plays a critical role, where epithelial cells are reorganized into mesenchymal cells that secrete ECM components [23]. Several other processes have also been indicated, including chronic inflammatory responses, stress responses, and mitochondrial dysfunction [5]. These hallmarks are utilized in the studies below as metrics for evaluating the extent and progression of fibrosis.

### 2.3. Novel Insights for Translational Regulation of Fibrosis

Fibrosis is often a consequence of aberrant wound healing processes. From an evolutionary perspective, there is a clear need for a rapid response to injury to minimize damage. Rather than relying on the relatively slower transcriptional upregulation, one hypothesis is that the mRNAs encoding proteins related to wound healing and fibrosis are already present in the cells, but are not translated, or are translated with a low efficiency. After injury, signaling cascades may trigger dynamic RNA effectors that rapidly upregulate the translation of the sequestered pool of mRNAs within minutes. However, during chronic injury, disruption of these fine-tuning mechanisms, along with RNA transcript modifications, may lead to fibrosis [4].

## 3. Non-Coding RNAS in Translational Regulation of Fibrosis

### 3.1. Overview of Non-Coding RNAs

Non-coding RNAs (ncRNAs) are highly versatile RNA molecules that do not encode proteins, but play critical regulatory roles in cellular processes [24,25]. These include the well-known types, including transfer RNAs (tRNAs) and ribosomal RNAs (rRNAs), as well as microRNAs (miRNAs), long non-coding RNAs (lncRNAs), circular RNAs (circRNAs), small nucleolar RNAs (snoRNAs), small nuclear RNAs (snRNAs), and PIWI-interacting RNAs (piRNAs). Together, ncRNAs make up 85–90% of the total RNA in cells, while protein-coding RNAs (mRNAs) only account for 5–8% [26]. The dysregulation of ncRNAs is associated with a diverse range of diseases, including cardiac disorders [27], kidney diseases [28], liver diseases [29], cancers [30], and various others [24]. Although ncRNAs have been implicated in various cellular processes, their detailed functions are still being elucidated, especially in the context of IPF.

**Figure 2:**
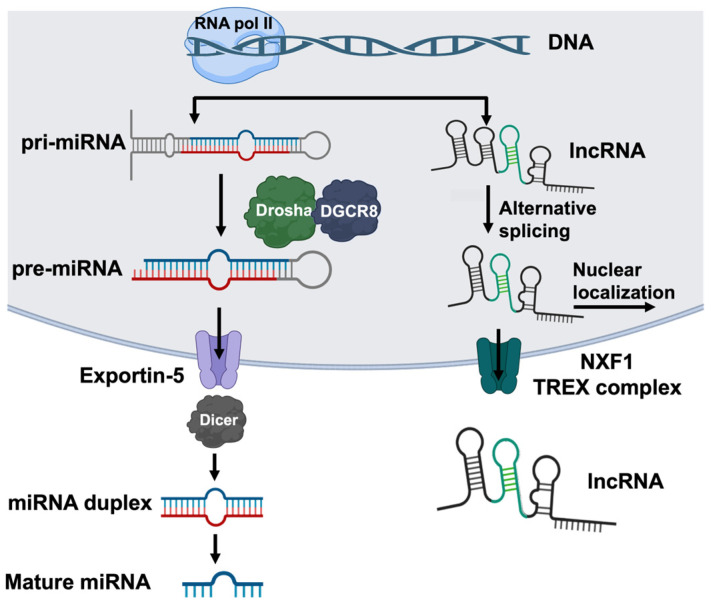
The biosynthesis of miRNAs and lncRNAs. miRNAs and lncRNAs are transcribed by RNA polymerase II, often from intronic or intergenic regions. The transcription of miRNA (left) produces a capped and polyadenylated primary microRNA (pri-miRNA) containing a characteristic hairpin and single-stranded RNA structure. The Drosha-DGSR8 complex cleaves the junction of the single-stranded RNA and hairpin to produce the precursor miRNA (pre-miRNA), which is exported to the cytoplasm via exportin-5. In the cytoplasm, Dicer further processes the pre-miRNA into 20–22-nucleotide double-stranded RNA duplexes that then split to form mature miRNA. The transcription of lncRNA (right) forms long transcripts that are capped, polyadenylated, and alternatively spliced. lncRNAs predominantly localize to the nucleus; however, a fraction of the molecules are exported via Nuclear RNA export factor 1 (NXF1) or the Transcription Export Complex (TREX). This figure was created using BioRender elements https://biorender.com/ (accessed on 6 August 2024).

miRNAs are short, 20–22-nucleotide-long regulatory molecules. They are transcribed by RNA polymerase II, often from intronic or intergenic regions, which results in a long capped and polyadenylated transcript called a primary microRNA (pri-miRNA), consisting of a hairpin structure and single-stranded RNA [31] (Figure 2). A ribonuclease complex, Drosha-DGCR8, recognizes the junction between the hairpin and the single-stranded RNA, cleaves the pri-mRNA, and forms a hairpin-shaped structure known as precursor miRNA (pre-miRNA), which is exported from the nuclear membrane through the exportin-5 channel [32]. In the cytoplasm, another ribonuclease, Dicer, processes the pre-miRNA into short, double-stranded RNA fragments, which separate and can adhere to the RNA-induced silencing complex (RISC) to target mRNA (Figure 2). miRNAs exert post-transcriptional regulatory effects by hybridizing to a specific seed region in target mRNA sequences, usually at the 3′UTR. miRNAs can be organized into clusters, which refer to miRNAs transcribed from physically adjacent genes, or families, which are miRNAs that bind to the same seed region, and may target the mRNAs encoding related proteins [33]. It is estimated that at least 30% of human genes are under miRNA regulation [34]. Additionally, one miRNA can regulate the expression of up to ~30 genes, and thus their impact may be profound [35]. miRNAs bound to their target mRNAs have been found in processing bodies (P-bodies), which are organelles in eukaryotic cells that contain the proteins and mRNAs involved in mRNA processing [36]. For some mRNAs normally found in the cytoplasm, miRNA binding causes the mRNAs to localize to the P-body [37]. Although miRNAs are most prominently known as inhibitors, in yeast, some miRNAs can either repress or stimulate translation, suggesting a more dynamic and environment-specific response [38,39]. Additionally, miRNA repression can be reversible depending on the cellular environment. For instance, in human liver cancer cells, the mRNAs encoding cationic amino acid transporter 1 (CAT-1) are repressed by miR-122 in a complex localized to P-bodies. Under stress conditions, the miRNA is released from the CAT-1 mRNA, the transcript is released from the P-body, and the translation of CAT-1 begins [40]. An intriguing possibility is that a similar miRNA-dependent pathway mediates the level of profibrotic proteins in response to chronic injury.

lncRNAs are typically around 200 nucleotides long and are processed similarly to mRNA, where they are transcribed, capped, polyadenylated, and alternatively spliced [31,41] (Figure 2). lncRNAs predominantly localize to the nucleus; however, a fraction of the molecules are exported from the nucleus via Nuclear RNA export factor 1 (NXF1) or the Transcription Export Complex (TREX) [42]. Due to their length, they can exhibit secondary structures which add another level of complexity in their regulatory activity. Although there are many potential mechanisms by which lncRNAs can regulate gene expression, our understanding of their interactions and specific binding is still primitive [41]. One of the better-understood mechanisms of their regulation is through their interactions with miRNAs, where the lncRNA competes with the target mRNA for miRNA binding, sequestering the miRNA [43,44,45]. This is the mechanism used by the lncRNAs that play a role in IPF, as discussed in this review.

circRNAs are a class of ncRNAs that are generated by an alternative splicing method called back-splicing, where the 3′ end of a transcript ligates to its own 5′ end, forming a covalently closed loop structure rather than the traditional linear structure [46,47]. This secondary structure also provides added stability, with circRNAs being generally more resistant to exonuclease degradation [48]. circRNAs have been observed across various organisms and are expressed in a wide range of tissues and cell types [46]. Although their formation mechanisms and cellular functions are not completely understood, circRNAs are made from non-coding regions and introns of coding genes, and can also be derived from exonic regions. This diversity allows for multiple mechanisms of regulation. For example, circRNAs derived from non-coding regions can act as miRNA sponges, sequestering miRNA function. Those originating from exonic regions are generated at the expense of canonical mRNA isoforms, potentially regulating mRNA production [46]. Several circular RNAs have been implicated in IPF; however, these are not discussed in this review, as their roles and functions are discussed in detail in a recent review [49].

In addition to miRNAs, lncRNAs, and circRNAs, three other ncRNA classes (snoRNAs, snRNAs, and piRNAs) are less understood in the context of IPF, but may also play regulatory roles. snoRNAs are found in the nucleoli of eukaryotic cells and help regulate the chemical modifications of other RNA species, primarily rRNAs; however, their involvement in the broader regulation of gene expression is an area of ongoing investigation [26,50]. snRNAs are essential components of the spliceosome complex, facilitating intron removal during mRNA splicing and playing a role in ribosomal RNA processing [26,51]. Lastly, piRNAs interact with PIWI proteins to form complexes that target and silence transposable elements which could otherwise destabilize the genome. piRNAs help preserve genome integrity, particularly in germline cells [52].

**Figure 3:**
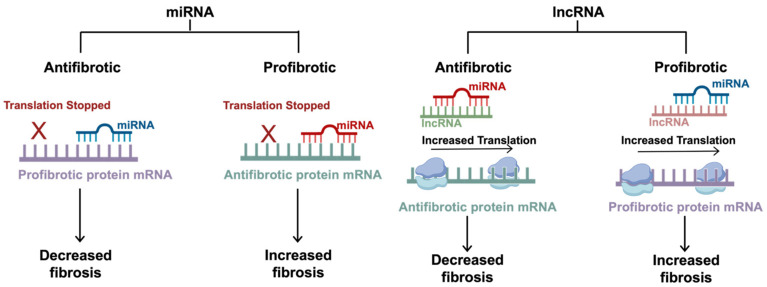
The mechanisms of non-coding RNAs in fibrosis. miRNAs are small regulatory RNAs, typically 20–22 nucleotides in length, that can induce antifibrotic or profibrotic effects depending on the target mRNA. miRNAs exert inhibitory effects by hybridizing to a specific seed region at the 3′UTR. Antifibrotic miRNAs target profibrotic proteins, and profibrotic miRNAs target antifibrotic proteins. lncRNAs are typically around 200 nucleotides long and regulate translation by sequestering the effects of the miRNAs, competing with the target mRNAs for miRNA binding. Antifibrotic lncRNAs target profibrotic miRNAs (which typically inhibit antifibrotic proteins), allowing for increased translation of the antifibrotic proteins. Similarly, profibrotic lncRNAs target antifibrotic miRNAs (which typically inhibit profibrotic proteins), allowing for increased translation of the profibrotic proteins. This figure was created using BioRender elements https://biorender.com/ (accessed on 6 August 2024).

Although all of the listed ncRNAs are involved in gene expression regulation and controlling various cellular processes, this review will primarily focus on the role of miRNAs and lncRNAs, with identified targets that are implicated in IPF. Comprehensive miRNA analyses using 1810 miRNA probes found 161 miRNAs that were differentially expressed in the lungs of mice with and without bleomycin-induced pulmonary fibrosis [53]. Instead of providing a comprehensive synopsis of these 161 miRNAs, this section discusses 21 of the 161, focusing on those where a direct target in the fibrotic pathway was identified. Additionally, four lncRNAs that are prominent in IPF with known targets are discussed. Since many of the ncRNAs affect, or are a part of, the TGF-β1 signaling cascade, the sections below will discuss the miRNAs based on the following categorization: profibrotic/antifibrotic and canonical/noncanonical. In this context, “canonical” refers to the miRNAs that directly play a role in regulating fibrosis within the canonical TGF-β1/SMAD signaling cascade, whereas “noncanonical” refers to all other related pathways that may lie upstream, downstream, or outside of the TGF-β1/SMAD pathway. The general mechanisms of the ncRNAs are outlined in Figure 3, and their specific functions are summarized in Table 1.

### 3.2. Antifibrotic Canonical miRNAs

Antifibrotic canonical miRNAs inhibit the translation of the mRNAs encoding proteins that are directly involved in the TGF-β1/SMAD pathway. For instance, miR-326 targets the 3′UTR of the TGF-β1 mRNA itself and inhibits its expression [54]. miR-326 is downregulated in patients with IPF, and the intranasal delivery of miR-326 in mice reduces βbleomycin-induced lung fibrosis [55]. There are also multiple miRNAs that target the mRNAs encoding the TGF-β1 receptor, a dimer made up of TβRI and TβRII [56]. miR-26a specifically targets TβRI [57], while miR-9-5p [58,59], miR-18a-5p [60], the miR-19a-19b-20a subcluster [61], and miR-153 [62,63] target TβRII. miR-1343 is non-specific and targets both TβRI and TβRII [64]. In both mice with bleomycin-induced pulmonary fibrosis as well as in human lung fibroblasts, the upregulation of these miRNAs alleviates fibrotic effects, and the downregulation or inhibition of the miRNA worsens fibrosis [56,57,58,59,60,61,62,63]. Additionally, other miRNAs specifically target SMAD mRNAs. miR-486-5p [65,66] and miR-323a-3p [67] inhibit SMAD2, miR-29 inhibits SMAD3 [68,69], and miR-27a-3p inhibits both SMAD2 and SMAD4 [70]. Lastly, along with targeting the TGF-β1 receptor, miR-26a also inhibits SMAD4, which halts the nuclear translocation of *p*-SMAD3 and inhibits downstream fibrotic regulators [43].

### 3.3. Antifibrotic Noncanonical miRNAs

Antifibrotic noncanonical miRNAs exert antifibrotic effects which are either not closely associated with the TGF-β1/SMAD pathway or are upstream or downstream of this pathway. The miR-17~92 cluster is a polycistronic transcript with six miRNAs: miR-17, miR-18a, miR-19a, miR-19b, miR-20a, and miR-92a [71,72]. This cluster plays a key role in lung homeostasis and regulates the expression of various profibrotic genes, including metalloproteinases, collagen, and growth factors. There is an epigenetic feedback loop between the miR-17~92 cluster and DNA methyltransferase-1 (DNMT-1) expression where DNMT-1 is overexpressed in IPF patients, inducing the epigenetic silencing of miR-17~92, which normally regulates DNMT-1 expression [73]. This leads to the unregulated production of the profibrotic genes regulated by this cluster. Adding the miRNA cluster to IPF lung fibroblasts reduces DNMT-1 expression, reduces the expression of profibrotic genes, and normalizes the fibroblast phenotype [73]. The miR-19a, -19b, -26b subcluster inhibits CTGF expression in lung fibroblasts [7]. Interestingly, the miR-19a-3p/19b-3p cluster also inhibits autophagy in human cardiac fibroblasts by targeting TβRII [74]. The overexpression of this miRNA subcluster decreases the severity of pulmonary fibrosis in a bleomycin mouse model [7]. miR-27b is another antifibrotic effector, and TGF-β1 downregulates its levels. In human lung epithelial cells, miR-27b binds and inhibits the translation of the mRNA encoding gremlin-1, a profibrotic agent that is induced by TGF-β1 [75]. miR-155 inhibits at least two targets: (1) the mRNA encoding liver X receptor (LXR*α*), whose signaling exacerbates lung fibrosis [76], and (2) the mRNA encoding keratinocyte growth factor (KGF), which is primarily involved in the EMT response [77]. In addition to its role in the canonical pathway inhibiting TβRI and SMAD4 [43,78], miR-26a also targets high mobility group protein A2 (HMGA2), which is a key factor in EMT and is upregulated in IPF [79,80]. HMGA2 is regulated by 33 other miRNAs, including let-7d, which is downregulated in the lungs of IPF patients [81] Lastly, along with its canonical role in targeting SMAD3 [69], miR-29 also targets the mRNA encoding type I collagen (COL1A1), which is a key component of scar tissue [82].

### 3.4. Profibrotic Canonical miRNAs

The one well-described profibrotic canonical miRNA is miR-21, which promotes TGF-β1 signaling by inhibiting the expression of the negative regulator SMAD7 [83,84]. In alveolar epithelial cells, TGF-β1 upregulates the levels of miR-21 and causes EMT [84]. miR-21 shows increased expression in the lungs of bleomycin-treated mice, as well as the lungs of IPF patients [84,85]. Conversely, the inhibition of miR-21 in the mouse bleomycin model of pulmonary fibrosis prevented the increased expression of the fibrotic markers vimentin and smooth muscle actin (*α*-SMA) and attenuated EMT [84]. Interestingly, in cardiac fibrosis, miR-21 upregulation may be protective early after myocardial infarction, but detrimental in later stages. Specifically, the overexpression of miR-21 reduced the myocardial infarct size and left ventricular dimensions [86,87]. However, the potentially protective effects of miR-21 have not been tested in IPF models.

### 3.5. Profibrotic Noncanonical miRNAs

Profibrotic noncanonical miRNAs inhibit negative regulators of fibrosis that are not directly in the TGF-β1/SMAD pathway. miR-424 targets SMAD ubiquitin regulatory factor 2 (SMURF2), which inhibits TGF-β1 signaling by ubiquitination of the TGF-β1 receptors and SMAD proteins [88,89]. miR-199a-5p is another profibrotic mediator that binds the mRNA encoding caveolin-1 (CAV1), which normally inhibits TGF-β1 signaling by internalizing and degrading the TGF-β1 receptor [90,91]. miR-145 exerts profibrotic effects by inhibiting the expression of the transcription factor Krüppel-like factor 4 (KLF4) [92]. KLF4 interferes with the binding of SMAD2/3 to the α-SMA promoter [93,94]. When miR-145 inhibits the KLF4 expression, there is an increase in the α-SMA expression and exacerbation of the pulmonary fibrosis [94]. miR-145 also promotes the release of active TGF-β1 from LAP, but the mechanistic details of this are unknown [94]. Lastly, miR-215-5p represses the expression of bone morphogenetic protein receptor 2 (BMPR2), which inhibits the TGF-β1 signaling pathway by targeting SMAD3 [95]. The knockdown of miR-215-5p in a paraquat-induced pulmonary fibrosis model decreased the severity of fibrosis [96].

### 3.6. lncRNAs in Idiopathic Pulmonary Fibrosis

lncRNA-PFI is an antifibrotic RNA that is downregulated in the alveolar epithelial cells of mice with bleomycin-induced lung fibrosis [44]. lncRNA-PFI binds and sequesters miR-328-3p, an miRNA that exacerbates lung injury by inhibiting anti-apoptosis genes [44]. lncRNA-PFI also has an antifibrotic effect by inhibiting the expression of serine/arginine splicing factor 1 (SRSF) [97], but the details of this pathway require further investigation.

lncRNA-PCF is a profibrotic RNA that sequesters miR-344a-5p [45]. miR-344a-5p inhibits the effects of mitogen-activated protein kinase kinase kinase 11 (MAP3K11), which is a profibrotic mediator [45]. Similarly, lncRNA-PFAR (pulmonary fibrosis-associated RNA) is upregulated in fibrotic lungs, and promotes fibrogenesis by inhibiting miR-138. miR-138 targets the yes1-associated transcriptional regulator (YAP1), which is a profibrotic transcription coregulator that promotes cell proliferation, migration, and collagen production in lung fibroblasts, and specifically plays a role in IPF [98,99].

lncRNA-snhg6 is a profibrotic RNA that sequesters miR-26a [43]. As mentioned above, miR-26a downregulates TβRI interaction with TβRII, targets SMAD4 signaling, and downregulates HMGA2-induced EMT, thus exerting antifibrotic effects. lncRNA-snhg6 is upregulated in the lungs of mice with bleomycin-induced pulmonary fibrosis, and the knockdown of lncRNA-snhg6 alleviates pulmonary dysfunction in this model [43].

**Table 1:** List of miRNAs, lncRNAs, and their targets and functions in idiopathic pulmonary fibrosis.

Name	Category	Target	Function	Reference
miR-326	Antifibrotic Canonical	TGF-β1	Downregulates the TGF-β1-induced activation of the canonical and noncanonical pathways and upregulates SMAD7.	[54,55]
miR-26a		TβRI and SMAD4	Downregulates TβRI receptor interaction with TbRII and inhibits the nuclear translocation of p-SMAD3 by targeting SMAD4	[43,57]
miR-9-5p		TβRII	Downregulates TGF-β1 signaling	[58,59]
miR-18a-5p		TβRII	Downregulates TGF-β1 signaling	[60]
miR-19a, -19b, -20a		TβRII	Downregulates TGF-β1 signaling	[61]
miR-153		TβRII	Downregulates TGF-β1 signaling	[62,63]
miR-1343		TβRI and TβRII	Downregulates TGF-β1 signaling	[64]
miR-486-5p		SMAD2	Inhibits downstream SMAD-dependent effector molecules	[65,66]
miR-323a-3p		SMAD2	Inhibits downstream SMAD-dependent effector molecules	[67]
miR-29		SMAD3	Inhibits the binding of the SMAD3/SMAD2/SMAD4 complex to the target mRNA and inhibits downstream SMAD-dependent effector molecules	[68,69]
miR-27a-3p		SMAD2 and SMAD4	Inhibits downstream SMAD-dependent effector molecules	[70]
miR-17~92	Antifibrotic Noncanonical	DNMT-1	Inhibits profibrotic genes and has an epigenetic feedback loop with DNMT-1 to regulate the profibrotic genes	[73]
miR-19a, -19b, -26b		CTGF	Inhibits downstream ET-1 and thrombin profibrotic effects	[7]
miR-27b		Gremlin-1	Downregulates the profibrotic TGF-β1-induced Gremlin-1 signaling cascade	[75]
miR-155		LXRα and KGF	Downregulates the profibrotic effects of LXRα and KGF-induced EMT	[76]
miR-26a		HMGA2	Downregulates HMGA2-induced EMT	[78,79]
let-7d		HMGA2	Downregulates HMGA2-induced EMT	[81]
miR-29		COL1A1	Inhibits TGF-β1-induced collagen 1 upregulation	[82]
miR-21	Profibrotic Canonical	SMAD7	Inhibits the antifibrotic effects of SMAD7, the TGF-β1 canonical pathway inhibitor	[84]
miR-424	Profibrotic Noncanonical	SMURF2	Inhibits the antifibrotic regulation of TGF-β1-induced EMT	[89]
miR-199a-5p		Caveolin-1	Inhibits the antifibrotic regulation of TGF-β1 signaling	[91]
miR-145		KLF4	Inhibits the antifibrotic regulation of α-SMA	[92,94]
miR-215-5p		BMPR2	Inhibits the antifibrotic regulation of TGF-β1 signaling	[96]
lncRNA PFI	Antifibrotic Noncanonical	miR-328-3p	Inhibits the profibrotic effects of miR-328-3p	[44,97]
lncRNA-PCF	Profibrotic Noncanonical	miR-344a-5p	Inhibits the antifibrotic effects of miR-344a-5p	[45]
lncRNA-PFAR		miR-138	Inhibits the antifibrotic effects of miR-138	[99]
lncRNA-snhg6		miR-26a	Inhibits the antifibrotic effects of miR-26a	[43]

## 4. Transcript Modifications in Translational Regulation

### 4.1. Alternative Polyadenylation in Fibrosis

**Figure 4:**
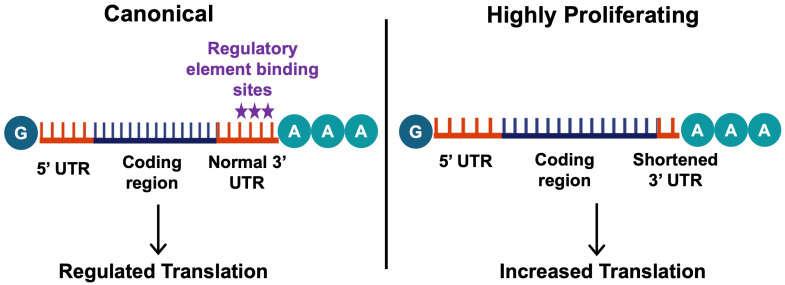
The mechanisms of alternative polyadenylation. Alternative polyadenylation involves differing polyadenylation sites, which influences the stability and level of translation of the transcript. In most healthy cells, the canonical distal polyadenylation site is preferred (left), leading to transcripts with long 3′UTRs allowing their translation to be regulated by various elements (stars indicate the common binding site of these elements). On the other hand, highly proliferating or differentiating cells prefer proximal polyadenylation sites, leading to 3′UTR shortening. This results in an increased translation of these transcripts, as the shorter mRNA variants often do not have translation-inhibiting elements in their 3′UTRs (right). This figure was created using BioRender elements https://biorender.com/ (accessed on 6 August 2024).

Along with the regulation of translation by the effector molecules, changes in the non-coding regions of mRNAs can also regulate translation and mediate the progression of disease. One of these modifications is alternative polyadenylation (APA), which influences the stability of the transcript [100]. This process plays a role in various cancers [101], and has also been associated with fibrosis [102]. Polyadenylation is the process of adding multiple adenosines to the 3′UTR of an mRNA, resulting in a polyA tail, which is crucial for mRNA stability, translation, and translocation [100]. The polyA tail is added downstream of the polyadenylation signal sequence, AAUAAA, which is recognized by the polyadenylation complex [103]. More than 70% of human genes contain multiple polyadenylation sites, which result in mRNA transcripts with varying 3′UTR lengths known as alternative polyadenylation [104]. The distal polyadenylation site is usually preferred in healthy cells, which leads to transcripts with long 3′UTRs allowing them to be regulated by various elements such as miRNAs. However, highly proliferating or differentiating cells prefer proximal polyadenylation sites, which lead to 3′UTR shortening. A shorter 3′UTR results in robust protein expression, as the shorter mRNA variants often do not have translation-inhibiting elements in their 3′UTRs [105] (Figure 4).

The choice of the proximal or distal polyadenylation site is determined by interactions between many polyadenylation regulatory factors. There are 20 known core proteins that make up the following four polyadenylation complexes: 1. cleavage and polyadenylation specificity factor (CPSF), 2. cleavage stimulation factor (CstF), and 3. and 4. cleavage Factors I and II (CFIm and CFIIm) [104]. Cleavage factor Im25 (CFIm25, also known as Nudix hydrolase 21, NUDT21), is a key component of the CFIm complex and plays a role in polyadenylation site regulation, with a preference for the distal site [106]. The downregulation of CFIm25 induces the widespread shortening of mRNA 3′UTRs, leading to an increase in the levels of the corresponding proteins [105]. Notably, the downregulation of CFIm25 enriches profibrotic pathways including TGF-β1 and the wingless-related integration site (WNT) [106]. TGF-β1 upregulates miR-203, which binds to and inhibits the mRNA encoding CFIm25, thus depleting CFIm25 and further potentiating fibrosis [102]. CFIm25 is downregulated in the lungs of IPF patients and bleomycin-treated mice, and the knockdown of CFIm25 caused the upregulation of the fibrotic markers collagen I (COL1A1) and fibronectin in healthy lung fibroblasts and exacerbated fibrosis in bleomycin-treated mice [106].

Another polyadenylation site selection factor that has been implicated in fibrosis is cleavage stimulatory factor 64 (CstF64). The CstF complex interacts with the conserved G/U-rich sequences downstream of the polyadenylation signal and helps determine the length of the 3′UTR. In contrast to CFIm25, CstF64 favors polyA addition at the proximal polyadenylation site. As a result, CstF64 induces mRNA 3′UTR shortening and increases protein expression [107]. The levels of CstF64 are upregulated in the left ventricle of heart failure patients and in a cardiac fibrosis model, and the increased expression of CstF64 shortened the 3′UTRs of the mRNAs encoding α-SMA, collagen 1, fibronectin, TGF-β1, and TβRI, leading to increased levels of these proteins and increased cardiac fibrosis [107]. Conversely, the depletion of CstF64 led to increased 3′UTR lengths of the mentioned profibrotic mRNAs, decreasing the levels of the corresponding fibrosis-associated proteins [107].

Overall, these studies indicate that alternative polyadenylation factors can function upstream, downstream, and outside of the TGF-β1 signaling cascade to affect the translational regulation of fibrosis. Although current studies demonstrate the widespread regulation of mRNAs, there may be fibrosis-specific regulatory elements that influence alternative polyadenylation factors which are differentially stimulated during chronic injury. Further investigation is needed to uncover this possibility.

### 4.2. mRNA Chemical Structure Modifications

mRNA transcript chemical modification is another emerging aspect of fibrosis regulation. Modifications including the N^6^-methylation of adenosine (m^6^A), N^1^-methylation of adenosine (m^1^A), 5-methylcytosine (m^5^C), pseudouridine (Ψ), 7-methylguanosine (m^7^G), and adenosine to inosine (A-to-I) editing have been implicated in lung diseases, but the mechanisms have not been fully elucidated [108]. These modifications fine-tune gene expression, and thus they likely modulate fibrosis-related effector molecules. m^6^A is the most common mRNA modification in mammals, and fibrotic organs have an abnormal pattern of m^6^A. Specifically, the m^6^A modification is upregulated in a bleomycin-induced mouse model of pulmonary fibrosis and IPF patient lung samples, and lowering the m^6^A levels inhibits the key fibroblast to myofibroblast activation [109]. Additionally, in an air pollution (PM_2.5_)-induced model of pulmonary fibrosis, the fibrotic lung tissue exhibited an abnormal increase in mRNA m^5^C modifications, especially in transcripts involved in oxidative stress and inflammatory response [110]. Although these modifications have been correlated to the fibrosis pathway, there is still much to learn about the role of epigenetic RNA modifications in pulmonary fibrosis and their role in mediating downstream effectors.

## 5. Clinical Potential of RNA-Based Therapies for Fibrosis

While there are no specific therapies that target translational regulation for fibrosis, RNA-based therapies, including small interfering RNAs (siRNAs), antisense oligonucleotides (ASOs), and lncRNA binders, have demonstrated clinical success for cancers and metabolic disorders, offering a foundation for their potential application in fibrotic disorders. Specifically, siRNA therapies such as patisiran, givosiran, lumasiran, and inclisiran have been FDA-approved for hereditary transthyretin-mediated amyloidosis (hATTR), acute hepatic porphyria (AHP), primary hyperoxaluria type 1 (PH1), and heterozygous familial hypercholesterolemia (HeFH), respectively [111]. Similarly, ASO-based therapeutics have demonstrated potential in cancer, where the oligonucleotides that were complementary to the target downregulated aberrant miRNA activity [112,113]. Additionally, competitive binders like lncRNAs can be used to downregulate overexpressed endogenous miRNAs [114]. These advances suggest promising opportunities to apply similar strategies for fibrosis, where targeting profibrotic miRNAs with competitive inhibitors or supplementing antifibrotic miRNAs could help mitigate fibrotic progression.

While these successes underscore the feasibility of RNA-based therapies, the challenges include finding the therapeutic targets, finding the subsets of the targets where blocking or enhancing the target does not cause unwanted side effects, and finding the subset of these where there is no compensatory mechanism that will suppress the effects of the treatment [114]. Additionally, RNAs or RNA-like molecules are highly unstable; therefore, the standard difficulties of finding an effective therapeutic, including absorption/distribution/metabolism/excretion (ADME), need to be overcome. Recent advances in RNA delivery technologies, including the use of nanoparticles, exosomes, and micelles, have been made to help address these challenges [115,116,117]. The goal for these delivery systems is to create a tissue- and/or cell-specific delivery mechanism that can carry stable therapeutic RNAs/RNA-like molecules (siRNAs, miRNAs, ASOs).

Nanoparticles, commonly lipid-based, can be loaded with RNAs/RNA-like molecules using emulsion methods and administered into the bloodstream, where they are taken up by a target cell via endocytosis, delivering stable therapeutic molecules [115]. Although promising, multiple challenges remain, including high production costs, potential immune responses, and limited tissue penetration, specifically in dense tissues such as fibrotic lungs.

Exosomes are endogenous extracellular vesicles that offer another potential delivery mechanism. They have membranes that protect their cargo, including RNAs, from degradation, and exosomes can bind to specific surface receptors on target cells, facilitating targeted and rapid delivery [116]. Exosomes are critical for intracellular communication, immune responses, immune regulation, inflammation, and cell phenotype transformation, and play a role in IPF [118]. Due to these properties, exosomes can be targeted for degradation or engineered to deliver specific cargo, such as therapeutic RNAs or RNA-like molecules. Notably, macrophage-derived exosomes were engineered to deliver specific miRNAs, and were shown to alleviate pulmonary fibrosis in a bleomycin-induced mouse model [118]. Because exosomes are endogenous, they are less likely to provoke an immune response, and macrophage-derived exosomes can alleviate fibrosis by delivering specific miRNAs [119].

Micelles are amphiphilic molecules, i.e., molecules with both hydrophilic and hydrophobic components, that self-assemble into core shell structures in aqueous environments, and can also be used for drug delivery [117]. A recent study investigated RNA-loaded micelles that were nebulized for lung fibrosis therapy. This study loaded a siRNA for matrix metalloproteinase 7 (MMP7) (known to be upregulated in IPF) into a micelle that was nebulized, and showed that the application of the nebulized micelle to precision-cut lung slices decreased the MMP7 expression [117].

Along with RNA-based therapies, there is an emerging interest to target RNA modifications. Recent advancements in protein screening methods, such as ribosome profiling, mass spectrometry-based proteomics, and single-cell translation profiling, have enhanced our understanding, providing tools to identify and address the complexity of RNA translation regulatory networks and guide the development of more precise therapeutic strategies [120]. Current approaches to target modifications primarily involve m^6^A-methylation, and focus on maintaining or altering the balance between methylation and demethylation, especially in the context of cancer. For instance, small molecule inhibitors have been developed that target the fat mass and obesity-associated protein (FTO) responsible for erasing m^6^A modifications. These FTO inhibitors have shown promise in cancer, and could potentially be adapted for fibrotic conditions [121]. However, many challenges remain due to the absence of a specific, targetable methylation pattern and the broad range of genes that could be targeted. Therefore, further investigation is necessary to utilize this potential therapeutic route.

Overall, RNA-based strategies hold great potential for their application in treating fibrosis. Their successful application will require continued research to address delivery obstacles, decipher complex RNA regulatory networks, and gain a better understanding of the RNA dynamics in fibrosis.

## 6. Discussion

Translational regulation is a critical, and often overlooked, level of gene expression control in the development of fibrotic disorders. This review specifically highlights the role of microRNAs (miRNAs), long non-coding RNAs (lncRNAs), and RNA transcript modifications in pulmonary fibrosis. The diversity of the regulators involved in fibrosis is fascinating and reveals the complexity of fibrosis regulation. The role of individual effectors in modulating fibrosis raises questions about whether all regulators are required, if only some are sufficient, if they are interconnected, and if the downstream pathways activated by each regulator are the same. Further, it is unclear whether the fibrosis pathway is sequential where each effector is interdependent, or if there are only a few required signals and many downstream elements that can only potentiate or attenuate the level of fibrosis. While this review focused primarily on the TGF-β1 pathway, many other cytokines are involved in fibrosis, and their associated translational regulators require further investigation.

Future research will uncover how these effectors act in concert, identify which components are upstream versus downstream, determine if epigenetic RNA modifications can be specific for fibrosis, and importantly, identify any key processes or molecules that can be targeted therapeutically or as diagnostic biomarkers. Epigenetic RNA modifications including m^6^A, m^1^A, m^5^C, m^7^G, and others may play a role in regulating fibrosis pathways; however, the mechanisms underlying these modifications and their precise influence remain unexplored. The specificity of these modifications in modulating fibrosis-related molecules is particularly interesting, and further research is needed to uncover these aspects. Overall, we are at an exciting turning point in understanding fibrosis. Addressing the current knowledge gaps with a comprehensive approach will ultimately aid in the development of novel therapeutics and diagnostic tools for fibrotic disorders.

## Data Availability

As this is a review, there are no data associated with this paper.

## Abbreviations

The following abbreviations are used in this manuscript:

IPF	Idiopathic pulmonary fibrosis
miRNA	MicroRNA
lncRNA	Long non-coding RNA
ECM	Extracellular matrix
RBPs	RNA-binding proteins
TGF-β	Transforming growth factor beta
PDGF	Platelet-derived growth factor
CTGF	Connective tissue growth factor
TNF	Tumor necrosis factor
WNT	Wingless-related integration site
FGF	Fibroblast growth factor
EMT	Epithelial–mesenchymal transition
IL	Interleukin
INF	Interferon
ROS	Reactive oxygen species
MMP	Matrix metalloproteinase
TIMP	Tissue inhibitor of matrix metalloproteinase
VEGF	Vascular endothelial growth factor
LAP	Latency-associated peptide
RGD	Arginine-glycine-aspartic acid
NEU3	Neuraminidase 3
TβRI	Transforming growth factor beta receptor 1
TβRII	Transforming growth factor beta receptor 2
SMAD	Small mothers against decapentaplegic
MAPK	Mitogen-activated protein kinase
PI3K	Phosphatidylinositol 3-kinase
JNK	c-Jun N-terminal kinase
ERK	Extracellular signal-related kinase
RAS	Rat sarcoma virus
RAF	Rapidly accelerating fibrosarcoma
P-bodies	Processing bodies
CAT-1	Cationic amino acid transporter 1
LXRα	Liver X receptor
KGF	Keratinocyte growth factor
HMGA2	High mobility group protein A2
COL1A1	Type 1 collagen
SMURF2	SMAD ubiquitin regulatory factor
CAV1	Caveolin-1
KLF4	Krüppel-like factor 4
BMPR2	Bone morphogenetic protein receptor 2
SRSF	Serine/arginine splicing factor 1
MAP3K11	Mitogen-activated protein kinase kinase kinase
PFAR	Pulmonary fibrosis-associated RNA
YAP1	Yes1-associated transcriptional regulator
APA	Alternative polyadenylation
CPSF	Cleavage and polyadenylation specificity factor
CstF	Cleavage stimulation factor
CF	Cleavage factor
m^6^A	N^6^-methylation of adenosine
m^1^A	N^1^-methylation of adenosine
m^5^C	5-methylcytosine
m^7^G	7-methyguanosine
A-to-I	Adenosine to inosine
PM_2.5_	Particulate matter diameter of 2.5 μm or less
siRNA	Small interfering RNA
hATTR	Hereditary transthyretin-mediated amyloidosis
AHP	Acute hepatic porphyria
PH1	Primary hyperoxaluria type 1
HeFH	Heterozygous familial hypercholesterolemia
ADME	Absorption, distribution, metabolism, and excretion
FTO	Fat mass and obesity-associated protein
